# Exploring the Integration of Consumer Activity Trackers Into a Community Weight Management Intervention to Support Physical Activity in Adults at Risk for or With Type 2 Diabetes: Mixed Methods Study Using the RE-AIM Framework

**DOI:** 10.2196/91073

**Published:** 2026-03-25

**Authors:** William Hodgson, Alison Kirk, Marilyn Lennon, Xanne Janssen, David Kennedy

**Affiliations:** 1 Department of Psychological Sciences and Health, Physical Activity for Health Research Group, University of Strathclyde, 40 George Street, Glasgow, Scotland, G1 1QE, United Kingdom, 44 07753324172; 2 Department of Computer and Information Sciences, University of Strathclyde, Glasgow, Scotland, United Kingdom

**Keywords:** type 2 diabetes, physical activity, sedentary behavior, Fitbit, activity tracker, adults, Weigh to Go, weight management, RE-AIM framework, Reach, Effectiveness, Adoption, Implementation, and Maintenance

## Abstract

**Background:**

Type 2 diabetes affects 483 million adults worldwide, with rising prevalence and an estimated 6 million premature deaths annually. Low physical activity is a key risk factor, while increased activity can reduce disease onset and improve metabolic health. Consumer activity trackers, when paired with behavior change strategies, have shown potential to increase physical activity among adults with type 2 diabetes.

**Objective:**

This study explored the integration of consumer activity trackers into a community-based weight management intervention to support physical activity in adults at risk for or living with type 2 diabetes.

**Methods:**

A mixed methods design was used to generate a comprehensive understanding of implementation. Participants were recruited during registration for “Weigh to Go,” a community-based weight management program in Lanarkshire, Scotland. Health care professionals delivering the intervention were recruited by email. Participants received a Fitbit Charge 5 to monitor daily steps and moderate-to-vigorous–intensity physical activity. Semistructured interviews were conducted with 10 participants and 10 health care professionals. Qualitative data were analyzed thematically, and quantitative analysis examined changes in recorded Fitbit activity data. Findings were interpreted using the Reach, Effectiveness, Adoption, Implementation, and Maintenance framework.

**Results:**

Daily steps increased significantly between week 1 and week 7 (mean difference 5345 steps; *P*=.002). Qualitative findings highlighted 5 themes. First, providing devices free of charge enhanced reach by removing financial barriers. Second, educational classes were considered essential for effectiveness, particularly instruction on device use and interpretation of activity data. Third, staff expressed a need for greater understanding of device functionality and data outputs, supporting broader adoption of trackers within weight management services. Fourth, managers would benefit from a detailed protocol outlining tracker introduction, use, data analysis procedures, evaluation metrics, and costs to ensure efficient and consistent implementation. Fifth, extending compulsory attendance at intervention sessions was considered important for long-term maintenance of behavior change. The observed decline in moderate-to-vigorous–intensity physical activity after week 7 was attributed to challenges in sustaining engagement beyond the structured phase of the program.

**Conclusions:**

This study demonstrates the feasibility of integrating consumer activity trackers into a community-based weight management intervention. Applying the Reach, Effectiveness, Adoption, Implementation, and Maintenance framework revealed that free device provision, participant and staff education, clearly defined implementation protocols, and structured attendance expectations can strengthen tracker-supported interventions. The use of the Fitbit device as both a measurement and intervention tool also raises methodological considerations, emphasizing the need for future research to differentiate these dual roles. Overall, activity trackers show promise for supporting physical activity among adults at risk for or with type 2 diabetes when embedded within well-designed community programs. These findings underscore the importance of aligning technological tools with supportive behavioral strategies to maximize health outcomes. The results also highlight opportunities for refining community programs through closer integration of digital health tools.

## Introduction

Type 2 diabetes mellitus is a chronic noncommunicable disease that occurs when the body cannot produce enough insulin, which facilitates the uptake of glucose into cells. This, in turn, raises blood glucose levels. If left untreated, type 2 diabetes can lead to premature death. Complications of type 2 diabetes include a higher risk of developing cardiovascular disease, retinopathy, neuropathy, and nephropathy [1]. Globally, it is estimated that 483 million adults (aged 20‐79 years) are living with type 2 diabetes, and by 2045 this number is expected to rise to 700 million [2]. In the United Kingdom, 5 million adults have been diagnosed with type 2 diabetes, and their treatment costs the National Health Service £12 billion each year (or US $16.8 billion each year; conversion rate: £1=US $1.34) [3]. Major risk factors for developing type 2 diabetes are physical inactivity, sedentary behavior, and being overweight or obese [1]. Adults diagnosed with type 2 diabetes have been shown to be less physically active and spend more time being sedentary compared with those who do not have the disease [4]. The American Diabetes Association has stated that physical activity can help prevent adults from developing type 2 diabetes, and for those already diagnosed with the disease, it can improve their diabetes and general health [5]. Even low-intensity physical activity and a reduction in sedentary behavior can bring health benefits for adults diagnosed with type 2 diabetes by lowering their blood glucose levels and BMI [6,7].

Physical activity interventions can significantly reduce the blood glucose levels of adults diagnosed with type 2 diabetes [6-8]. Physical activity interventions that incorporate wearable technology, such as consumer activity trackers and pedometers, have been developed for adults diagnosed with chronic noncommunicable diseases, including those living with type 2 diabetes [9]. Consumer activity trackers, including Fitbit models, are valid and reliable devices for measuring users’ daily steps and physical activity intensity [10]. Physical activity interventions that incorporate a consumer activity tracker have been shown to lower blood glucose levels, reduce BMI, increase activity, and reduce sedentary time in adults living with type 2 diabetes [11]. Physical activity interventions that combine an activity tracker with either a behavioral change intervention or a web-based platform can bring significant health improvements for patients with type 2 diabetes [12]. In addition, consumer activity trackers have the potential to allow health care professionals to remotely monitor individuals’ physical activity as part of their clinical care and for the person to self-manage their activity through feedback from the device [13]. Community-based weight management interventions are 1 way to try and support the use of activity trackers by overweight adults and promote physical activity in this population [14]. The Weigh to Go program is an established community- based weight management intervention that aims to support adults to increase physical activity and improve diet. However, referral and adherence of adults with type 2 diabetes to the program have been low, and there has been limited evaluation of longer-term physical activity behavior change [14]. Integrating consumer activity trackers into such programs may help address these gaps by enhancing engagement, supporting self-monitoring, and providing continuous feedback. A further methodological consideration is that the activity tracker functions simultaneously as a measurement tool and an intervention component. This dual role can influence participant behavior through feedback, goal setting, and accountability features, meaning that the act of measurement may itself contribute to behavior change [13]. Recognizing this overlap is important for interpreting outcomes and for designing future studies that aim to disentangle these effects.

This study aimed to explore, through a mixed methods study using the Reach, Effectiveness, Adoption, Implementation, and Maintenance (RE-AIM) framework, the integration of consumer activity trackers into a community weight management intervention to support physical activity in adults at risk for or with type 2 diabetes. The RE-AIM framework was used not only as an evaluative structure but also as a sensitizing guide for data collection, enabling exploration of how the intervention reached participants, how it was experienced, how staff adopted and delivered it, and how it might be sustained in routine practice.

## Methods

### The Study Context: Weigh to Go Intervention

The Weigh to Go intervention is an established community-based weight management behavior change program based in Lanarkshire, Scotland. Sessions were held within 12 local community centers and sports centers. The intervention consists of a 15-week active phase and a 15-week maintenance phase. In the active phase, participants receive a 45-minute educational workshop and a 45-minute circuit-based physical activity session each week. Each session has been developed to stand alone, and evidence-based content has been developed by dietitians, nurses, and psychologists [14]. Sessions include discussion and reflection on dietary education content, as well as activities to support positive and sustainable behavior change through motivation-, action-, and prompt-informed health behavior change strategies [15]. In the maintenance phase, participants receive a short session with reflection on what was covered during the equivalent week in the active phase, alongside 45 minutes of physical activity. Attendance at the maintenance sessions is optional, whereas attendance at the active phase classes is expected. The standard physical activity component is circuit-based, with adaptations to accommodate most levels of fitness and mobility. The intervention is free for participants and is delivered in local leisure centers and community facilities. The Weigh to Go intervention has the potential to be an effective community-based program to support weight management and physical activity for adults with type 2 diabetes and those at high risk of developing the disease. However, referral and adherence of adults with type 2 diabetes to the Weigh to Go intervention have been low, and there has been limited evaluation, particularly of longer-term physical activity behavior change. Health care professionals also report limited follow-up on the progress of people they have referred [14].

### Justification for the Addition of Activity Trackers Into the Weigh to Go Program

In weight management programs such as Weigh to Go, client referral and sustained adherence are crucial determinants of success. Yet, traditional interventions relying primarily on dietary advice and tracking, with limited assessment of physical activity, often face high dropout rates [16]. The integration of wearable activity trackers could address these gaps and support self-monitoring of physical activity, behavior change, and ongoing engagement. Patients referred to weight management services frequently disengage early; lack of ongoing feedback contributes to this attrition [17]. Wearables provide continuous self-monitoring, one of the most effective behavior change techniques, which has been shown to mediate weight loss. Moreover, users who consistently track physical activity lose significantly more weight than those with low adherence [18].

Wearables deliver critical behavior change strategies. Self-monitoring and goal setting can be achieved through step tracking and recording of active minutes, which enhances motivation and self-awareness [19]. Feedback and habit formation can be achieved through automated reminders to support long-term adherence [20]. Providing professional oversight through consultations has been shown to significantly boost physical activity outcomes [21]. Meta-analyses show that wearables improve activity levels (~1800 additional steps/d, 40 minutes walking/d) and reduce body weight (~1 kg) across clinical populations [17]. In overweight adults, activity tracker–inclusive interventions outperformed standard programs over ≤6 months, particularly among middle-aged and older adults [16]. Another meta-analysis reported moderate-to-large standardized increases in daily steps (standardized mean difference 0.54) and moderate-to-vigorous physical activity (standardized mean difference 0.47), with meaningful reductions in weight and BMI [22].

The Weigh to Go program typically secures referrals based on clinical or motivational criteria. However, post referral, clients often lose momentum [14]. Embedding wearables at the referral point could potentially increase immediate engagement by giving clients tangible metrics and daily goals and produce early positive results for participants. This could also encourage long-term adherence and provide continuous data to tailor individual support. Pairing trackers with professional guidance has the potential to create a multimodal intervention—diet, activity, technological feedback, and counseling—which yields better short-term outcomes than standard programs alone [16].

Incorporating physical activity trackers into the Weigh to Go program has the potential to address critical gaps in referral and adherence. They operationalize evidence-based behavior strategies, including self-monitoring, goal setting, and feedback, supported by robust meta-analytic evidence. When paired with professional oversight, trackers enhance engagement, mitigate dropout, and drive measurable improvements in activity and weight loss. This technology-enhanced model not only aligns with best practices in behavioral medicine but also leverages scalable tools to boost program effectiveness and sustainability.

### Participants

#### Adults Registered on the Community Weigh to Go Intervention

Participants were recruited via a study flyer at various venues hosting the registration stage of the Weigh to Go intervention. The initial inclusion criteria included adults aged >18 years (no upper limit set), diagnosed with type 2 diabetes or at risk of developing the disease, residing in the United Kingdom, having access to internet services, and being able to read and write in English. Exclusion criteria included not being diagnosed with type 2 diabetes or not being at risk of developing the disease, or having been advised by a health care professional not to undertake physical activity. Participants interested in taking part in the study were asked to email the research team. The study participant information sheet and consent form were uploaded onto the secure Qualtrics (Silver Lake) survey system. A link to this form was emailed to interested participants, and their consent was recorded by indicating “yes” on the Qualtrics consent form. Once consent was received, participants were emailed a link to a baseline questionnaire on the Qualtrics system. This questionnaire gathered demographic details, educational level, current activity tracker use, and health conditions.

#### Health Care Professionals

Health care professionals (n=10) involved in the delivery and onboarding of the Weigh to Go intervention were recruited via an email from the research team. Recruitment criteria included adults aged >18 years, residing in the United Kingdom, and being a health care professional (eg, doctor, nurse, fitness instructor, or Weigh to Go intervention administrator). The study participant information sheet and consent form were uploaded onto the secure Qualtrics survey system. A link to this form was emailed to participants, and their consent was recorded. Once consent was received, a link to a Qualtrics questionnaire was emailed to the participant to gather information regarding demographics, qualifications, and job role characteristics.

### Procedure

**Figure 1.**
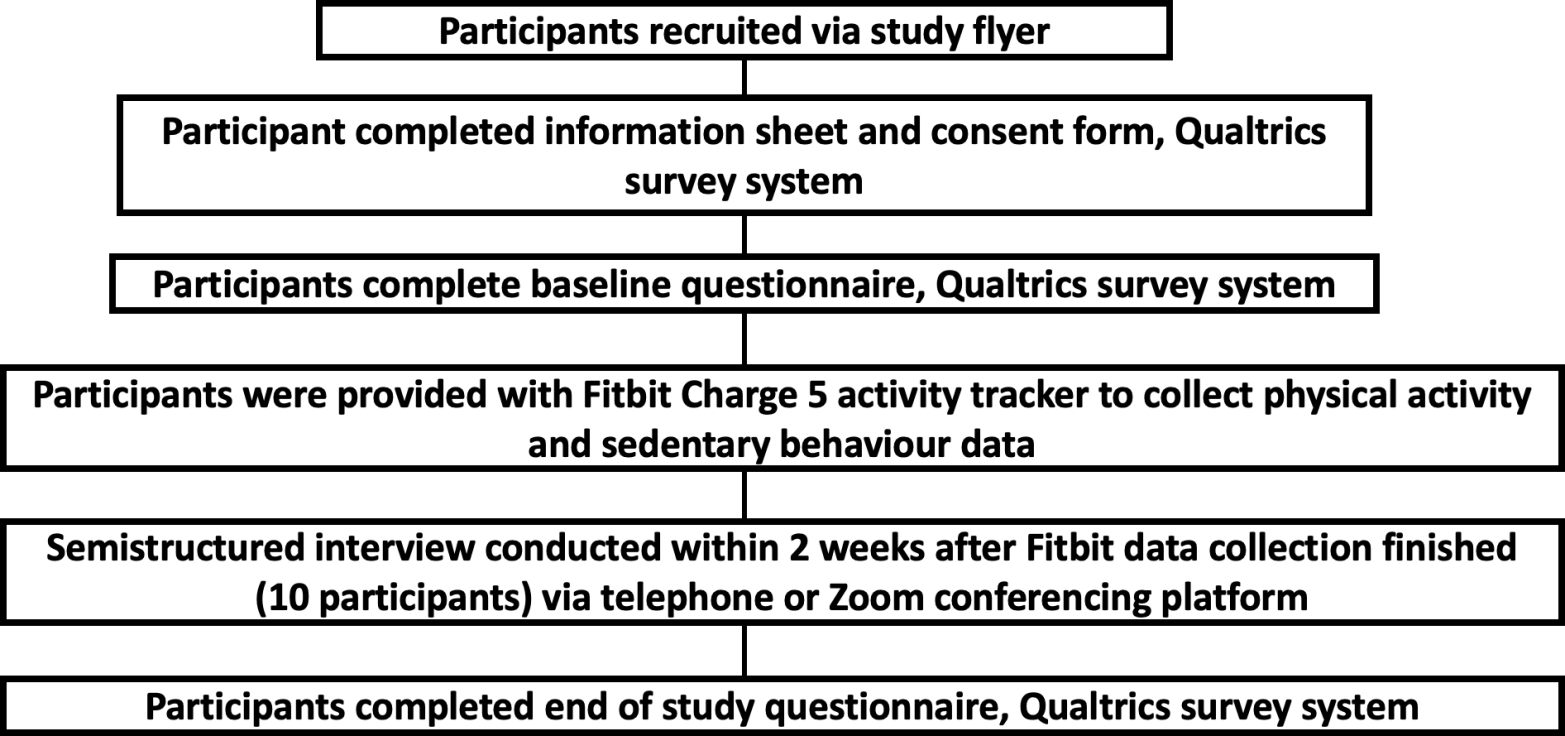
Flow diagram showing summary of the study procedures for the Weigh to Go intervention participants.

**Figure 2.**
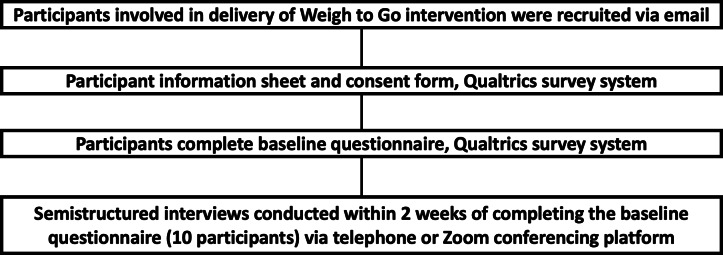
Flow diagram showing a summary of the study procedures for the Weigh to Go intervention for health care professionals.

A summary of the study procedures for the Weigh to Go intervention participants is provided in Figure 1. A summary of the study procedures for the Weigh to Go health care professionals is provided in Figure 2.

### Data Collection

#### Quantitative Data Collection

Once participants had consented and completed the baseline survey, they were provided with a Fitbit Charge 5 device (provided free of charge by Fitbit United Kingdom), which they were allowed to keep at the conclusion of the study. The research team set up an individual Fitbit account for each participant (Google Gmail email address and password) to allow both the participant and study team to access the activity data from the Fitbit device. Instructions on how to access this account and set up the Fitbit device were emailed to each participant. Additional support was provided, if required, via email, telephone, or face-to-face contact with the participants at the Weigh to Go venue. Participants were asked to use the Fitbit device over the period during which they attended the Weigh to Go intervention. Once each participant had finished attending the Weigh to Go intervention, they were sent an email containing a link to the Qualtrics end-of-study questionnaire. The research team downloaded the participants’ Fitbit activity data from the Fitbit account. The downloaded data included daily steps and weekly minutes of moderate-to-vigorous–intensity physical activity. The Fitbit data and the baseline and end-of-study questionnaire data were uploaded into SPSS (IBM Corp) statistical software for analysis.

#### Qualitative Data Collection

This study adopted a qualitative design to explore experiences of Fitbit use and engagement with the Weigh to Go intervention. The methodological orientation was constructivist and interpretivist, recognizing that knowledge is coconstructed through interaction and shaped by social and contextual factors [23,24]. Data were generated through semistructured interviews and analyzed using reflexive thematic analysis [25-27]. Reporting followed the COREQ (Consolidated Criteria for Reporting Qualitative Research) 32-item checklist, ensuring transparency across domains of research team and reflexivity, study design, and analysis [28].

A semistructured interview schedule was designed around the 5 RE-AIM dimensions [29]. These dimensions provided a structured yet flexible scaffold for exploring experiences of intervention use and delivery.

An abductive interviewing approach was adopted [30], enabling the researcher to attend to participants’ lived experiences while actively probing surprising, contradictory, or unanticipated insights. This iterative, dialogical style supported the generation of richer explanations by moving fluidly between empirical data and sensitizing theoretical concepts [31].

Interviews were conducted via secure Zoom (Zoom Video Communications) video conferencing or by telephone, based on participant preference. Each interview lasted between 45 and 90 minutes, was audio-recorded on an encrypted digital recorder, and then transcribed verbatim. Transcripts were anonymized, checked for accuracy, and uploaded to NVivo (version 12; Lumivero) for data management.

#### Study Design and Participants

One-to-one interviews were conducted with two groups: (1) ten Weigh to Go participants purposively sampled from those who provided Fitbit data, and (2) ten health care professionals drawn from the staff members involved in delivering the intervention.

Although participants were randomly selected within these eligible pools, the design was guided by a purposive sampling logic, ensuring recruitment of individuals directly engaged with Fitbit use or intervention delivery [24]. This approach balanced fairness in selection with the need for information-rich accounts. Interview questions were mapped to the RE- AIM dimensions. For example, “reach” questions explored barriers to joining or staying in the program; “adoption” questions asked staff about organizational readiness; “implementation” questions examined how trackers were introduced and supported; and “maintenance” questions invited participants and staff to reflect on whether and how tracker use could continue beyond the active phase. These questions focused on participants’ experiences and expectations rather than predictions of future behavior.

Sample size in qualitative research is not determined by statistical representativeness but by the adequacy of data to address research questions and generate meaningful insights [32,33]. The decision to recruit 20 participants in total was informed by methodological principles of information power, which suggest that smaller samples may be sufficient when study aims are specific, participants are closely aligned with the topic of inquiry, and the analysis is detailed [32]. Reflexive thematic analysis does not prescribe a fixed number of interviews; rather, it emphasizes depth, variation, and interpretive engagement over numeric thresholds [26]. Empirical benchmarks indicate that major thematic patterns can often be captured with 12‐15 interviews in relatively homogeneous samples [34,35], although professional perspectives typically add diversity and nuance [36]. Accordingly, this study adopted a pragmatic balance between analytic richness and feasibility, monitoring sufficiency throughout analysis and justifying the final sample size on grounds of analytic adequacy rather than data saturation as a fixed endpoint [26,33].

### Justification for Abductive Analysis

The study used abductive analysis because neither purely inductive nor deductive logic was sufficient for the research aims. Inductive approaches, while useful for grounded, bottom-up coding, risk limiting interpretation to surface-level description without connecting findings to broader explanatory frames [24]. Deductive approaches, by contrast, can overconstrain interpretation through rigid application of preexisting theories, missing novel, or contextually embedded meanings [37].

Abduction provided a middle ground, an iterative process of moving between empirical observations and theoretical frameworks [38]. In this study, abduction was particularly well suited because (1) surprise and anomaly were expected. Weigh to Go participants’ and health care professionals’ experiences with Fitbit use could not be fully anticipated, and abduction-supported theorizing around unexpected findings; (2) the RE-AIM framework informed but did not restrict analysis. Abduction enabled findings to dialog with RE-AIM concepts without being forced into them; and (3) the goal was to generate plausible explanatory insights. Abduction encourages researchers to adjudicate between rival explanations, ensuring that final themes represent the most coherent and plausible interpretations [31].

Thus, abduction was methodologically congruent with reflexive thematic analysis, which views researcher subjectivity and theoretical engagement as analytic resources rather than as sources of bias [26].

### Data Analysis

#### The Framework to Guide Analysis

The RE-AIM framework is a planning and evaluation tool used to improve the adoption and sustainable implementation of health-related interventions from research settings into real-world working environments [29]. The main RE-AIM dimensions are RE-AIM. Reach is defined as the absolute number, proportion, and representativeness of individuals participating in each initiative. Effectiveness is the impact of an intervention on outcomes, including potential negative effects, quality of life, and health components. Adoption is the proportion, representativeness, and absolute number of organizational agents involved in the intervention. Implementation is considered at an organizational level and refers to how the program was delivered by staff. Maintenance is defined as the extent to which a program or policy becomes embedded in routine practice [29]. At an individual level, maintenance is a measure of the long-term impact of an intervention over 6 months [39].

#### Quantitative Data Analysis

Descriptive analysis and repeated measures 1-way ANOVA tests were undertaken on the Fitbit data collected. One-way within-subjects ANOVA statistical tests were conducted to measure the effect of the participants’ use of the Fitbit Charge 5 activity tracker on their physical activity over 20 weeks. Magnitude of change was measured between week 1, week 7, week 15, and week 20 in relation to daily steps taken (number of steps) and weekly moderate-to-vigorous–intensity physical activity (minutes). The dependent variables were (1) mean daily step count and (2) mean weekly minutes of moderate-to-vigorous–intensity physical activity (MVPA). The independent variable was time, with 4 repeated measures corresponding to week 1, week 7, week 15, and week 20. This design enabled assessment of within-subject changes in physical activity across the intervention and immediate postintervention phases. Although the repeated-measures ANOVA assumes normally distributed data, inspection of residuals indicated that deviations from normality were minimal and within acceptable bounds for parametric testing. Given the moderate sample size, the ANOVA was considered robust to minor violations of normality. However, a nonparametric alternative (Friedman test) was also considered. In exploratory analyses, results from the Friedman test were consistent with the ANOVA findings, supporting the validity of the conclusions. Nonetheless, the parametric approach was retained as the primary method due to its greater interpretability and the ability to report effect sizes. The choice of time points (week 1, week 7, week 15, and week 20) was methodologically and practically driven. Week 1 acted as the initial reference point at the start of the intervention. Week 7 corresponded with the midpoint of the 15-week active phase, allowing assessment of early change and adherence. Week 15 marked the end of the active intervention phase, providing a natural point for evaluating intervention effects. Week 20 was included to assess whether physical activity behavior was maintained after the active phase concluded, aligning with the RE-AIM framework’s emphasis on maintenance. Data beyond week 20 (ie, weeks 25 and 30) were too sparse for valid statistical analysis and could have risked compromising participant anonymity.

#### Qualitative Data Analysis

Data analysis followed Braun and Clarke’s reflexive thematic analysis. NVivo 12 was used for organizing codes and data extracts, but interpretation remained the authors’ responsibility. Analysis was iterative and abductive, proceeding through Braun and Clarke’s six phases:

1. Familiarization: the author immersed himself in transcripts and audio, producing reflexive memos that captured first impressions, contradictions, and anomalies.
2. Generating initial codes: coding was conducted across the dataset at both semantic (descriptive) and latent (interpretive) levels. Abductive reasoning supported flexible refinement of codes when unexpected insights emerged.
3. Constructing candidate themes: codes were collated into candidate themes representing shared patterns of meaning relevant to the research question. Rival candidate structures were considered and abductively evaluated for explanatory power.
4. Reviewing and refining themes: candidate themes were tested against data extracts and the dataset as a whole. Incoherent or redundant themes were reworked, merged, or discarded. Negative cases were actively sought to challenge emerging interpretations.
5. Defining and naming themes: each theme was clearly defined with a central organizing concept and explanatory warrant. For example, the theme “Fitbit as accountability partner” captured both motivational and burdensome aspects of device use.
6. Producing the report: themes were written into an analytic narrative, weaving data extracts with interpretation to build plausible explanations that extended beyond description. Quotations were included as analytic evidence, not mere illustrations [26].

### Reflexivity and Rigor

In line with COREQ principles [28], reflexivity was integral throughout. Regular supervisory meetings provided reflexive dialog and guarded against premature closure of analysis. Trustworthiness was addressed using strategies congruent with reflexive thematic analysis [40,41]: (1) credibility through prolonged engagement with data, reflexive memoing, and analytic dialog; (2) dependability via a transparent audit trail documenting coding iterations and theme refinements; (3) confirmability by explicitly acknowledging researcher subjectivity as a resource in interpretation; and (4) resonance and contribution by producing findings with conceptual and practical utility for digital health intervention design.

### Ethical Considerations

Ethical approval for this mixed methods study was obtained from the University of Strathclyde Ethics Committee (approval no 2454). For the study, both adults diagnosed with type 2 diabetes and health care professionals were provided with web-based participant information sheets. Informed consent was obtained through the digital signing of a consent form. Participants electronically selected individual items in the digital form, corresponding to the paper consent form, to confirm that they had read and agreed to each item. Their electronic signature was achieved by entering their allocated 4-digit identification number. The research team undertook a number of steps to ensure the security of the information collected. To ensure anonymity, each participant was allocated a unique 4-digit identification number, and all data were stored under this number. Any information stored by the research team was stored within university-approved, password-protected, encrypted storage sites. Participants involved in this study received no compensation for taking part.

## Results

### Weigh to Go Participants Recruitment and Retention

**Figure 3.**
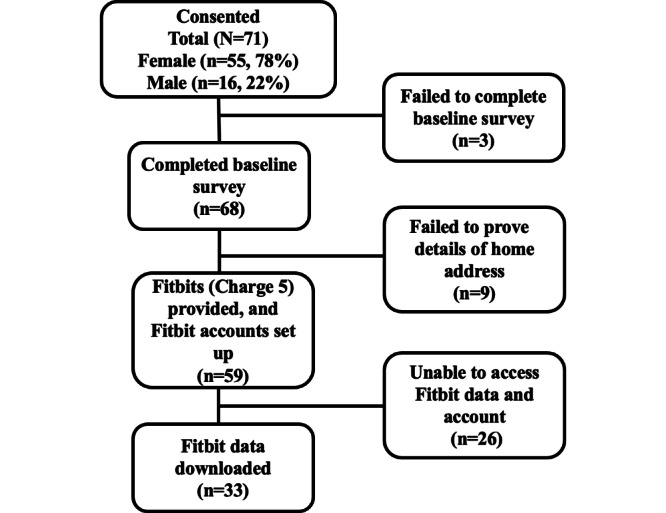
Weigh to Go participant recruitment and retention.

Weigh to Go participants were adults who had either volunteered to take part in the program or had been referred by a health care professional. Details of the Weigh to Go participant recruitment and retention are provided in Figure 3.

### Explanation of Fitbit Data Loss and Its Implications

In research involving digital health technologies, data integrity is paramount. Wearable activity trackers such as Fitbit devices offer researchers valuable opportunities to collect continuous, real-world data on physical activity. However, technological and procedural challenges can hinder data acquisition, leading to significant data loss. This was observed in this study, where Fitbit data loss affected both the completeness of analyses and the generalizability of findings.

Figure 3 illustrates participant recruitment and retention throughout the Weigh to Go intervention. Although participants were provided with Fitbit devices and accounts were set up by the research team, unexpected changes to Google’s security protocols during the study period caused substantial disruption. Specifically, midway through data collection, Google introduced additional 2-step verification processes for Fitbit accounts. This required participants to authorize access to their Fitbit data via a smartphone code linked to their Fitbit mobile app. Consequently, the research team could no longer directly access the accounts to download data.

To mitigate this, the team attempted to schedule telephone authorizations with participants during the download process. However, many participants (n=26) failed to respond to emails or were otherwise unavailable, which prevented access to their data. This issue ultimately resulted in the loss of approximately 26 (44%) participant Fitbit datasets, thereby contributing to the attrition shown in Figure 3. This data loss has implications for interpreting the quantitative findings, particularly regarding representativeness and the ability to assess integration across the full participant cohort.

### Participants Demographics

Weigh to Go participant and health care professional demographics are provided in Tables1  2. For the Weigh to Go participants, these included age, gender, educational qualifications, ethnicity, area of residence, health conditions, and previous use of an activity tracker. For the health care professionals, these included age, gender, educational qualifications, and ethnicity.

**Table 1.** Weigh to Go participant demographics.

Characteristic	Value
Age (years), mean (SD)	54.6 (12.24)
Sex, n (%)	
Male	6 (18)
Female	27 (82)
Educational qualifications, n (%)	
Degree	9 (27)
SVQ^a^	9 (27)
School qualifications	6 (18)
Higher education	7 (21)
Other qualifications	2 (7)
Ethnicity, n (%)	
White	33 (100)
Area of residence, n (%)	
Rural	3 (9)
Urban	30 (91)
Health conditions, n (%)	
Type 2 diabetes	17 (52)
Arthritis	9 (27)
Mental health	5 (15)
Asthma	3 (9)
COPD^b^	1 (3)
Stroke	1 (3)
Heart disease	1 (3)
Learning difficulties	1 (3)
Previous use of an activity tracker, n (%)	
Yes	1 (6)
No	31 (94)

a SVQ: Scottish Vocational Qualification.

b COPD: chronic obstructive pulmonary disease.

**Table 2.** Health care professional participant demographics.

Characteristic	Value
Age (years), mean (SD)	51.3 (9.7)
Sex, n (%)	
Male	3 (30)
Female	7 (70)
Educational qualifications, n (%)	
Degree	7 (70)
SVQ^a^	3 (30)
Ethnicity, n (%)	
White	9 (90)
Asian	1 (10)

a SVQ: Scottish Vocational Qualification.

### Quantitative Data Analysis

Results are presented under the appropriate RE-AIM main dimensions.

#### Reach

Seventy-one adults consented to take part in the study between July 2023 and January 2024. Of those who consented, 55 (78%) were female, and 16 (22%) were male. All 71 participants were White. We were unable to calculate the total number of Weigh to Go participants approached to take part in this study, as this was not recorded by the intervention staff. In total, 705 participants attended the Weigh to Go session venues targeted for this study (n=71, 10% of these consented to take part). The average number of participants attending each venue was 10.

#### Effectiveness

##### Weigh to Go Participants’ Fitbit Daily Steps (Number Taken)

A test of normality was carried out, and the assumption was met. The Mauchly test of sphericity produced a nonsignificant result (*P*=.62). Reporting sphericity assumed, there was a significant, large effect of Fitbit use on daily steps (*F*_3,45_=5.93; *P*=.002; η²=0.19). Post hoc comparisons using the Bonferroni correction for multiple comparisons were carried out. There was a significant increase in daily steps between week 1 and week 7 (*P*=.008), with participants in week 7 walking on average 5345 more steps. There was no statistically significant difference in steps between any of the other weeks.

##### Weigh to Go Participants Fitbit Weekly Minutes of Moderate to Vigorous Intensity Physical Activity

A test of normality was carried out and the assumption was met. Mauchly test of sphericity produced a significant result (*P*=.04). Reporting Greenhouse-Geisser correction showed that there was no significant effect of Fitbit use on weekly minutes of moderate-to-vigorous–intensity physical activity (*F*_1.68,13.45_=0.65; *P*=.51; η²=0.07). The decline in MVPA at weeks 15 and 20 reflects the transition from the structured active phase to the optional maintenance phase. Participants reported reduced accountability, fewer scheduled sessions, and diminished novelty of the device, all of which contributed to reduced engagement. This pattern suggests that the integration of trackers was most effective when embedded within structured program components, and less effective when participants were expected to self- manage without ongoing support.

This section evaluates how well the activity tracker was integrated into the program, rather than the program’s overall effectiveness. The observed changes in steps and MVPA illustrate how participants engaged with the tracker within the program context, highlighting the conditions under which integration supported behavior change.

### Adoption

The total number of staff involved in the delivery of the Weigh to Go intervention is provided in Table 3.

**Table 3.** Total number of staff involved in the delivery of the Weigh to Go intervention.

Role	Staff, n (%)
Intervention instructor	22 (55)
Project manager	8 (21)
Administration support	5 (12)
Researchers	5 (12)

The types and number of venues used for the delivery of the Weigh to Go intervention and the total number available throughout the 2 local councils are provided in Table 4.

**Table 4.** Types of venues used to deliver the Weigh to Go intervention.

Venue type	Number used, n (%)	Total number available, n
Community center	8 (7)	108
Community fitness center	4 (14)	29
Total	12 (9)	137

The adoption of the activity tracker varied across staff roles and settings. Fitness instructors and frontline staff were generally enthusiastic, viewing the tracker as a motivational tool that complemented existing program components. Administrative staff expressed uncertainty about their role in supporting device use, highlighting gaps in training and clarity.

Organizational adoption was influenced by (1) perceived relevance of trackers to program goals, (2) staff confidence in explaining device features, (3) availability of time during onboarding sessions, and (4) existing digital literacy among staff and participants.

Some staff described the tracker as an “add-on” rather than an integrated component, indicating partial adoption at the organizational level.

### Implementation

Signposting for the Weigh to Go intervention used by the study participants is provided in Table 5.

**Table 5.** Weigh to Go intervention signposting.

Signposting source	Value, n (%)
Diabetes nurse	5 (15)
Practice nurse	5 (15)
Local gym	5 (15)
GP^a^	3 (9)
Local community center	5 (15)
Website	4 (13)
Work email	1 (2)
Word of mouth	2 (7)
Facebook	2 (7)
Carer center	1 (2)

a GP: general practitioner.

The costs of delivering the Weigh to Go intervention included session cost per 15 weeks (US $672), session cost per 30 weeks (US $1344), venue cost per year (US $2016), annual intervention cost (US $60,480), average cost per participant per the 15-week active phase (US $67), and average cost per participant per the 30-week active and maintenance phase (US $134). Though the Fitbit devices were supplied free of charge for this study, the individual retail cost of purchasing the Fitbit Charge 5 was US $136.37. The total retail cost for this study would have been US $8046.03.

The mean number of sessions Weigh to Go participants attended during the 15-week active phase of the Weigh to Go intervention was 11.35 (SD 3.63) sessions.

#### Fidelity of Delivery

Implementation fidelity varied across sites. Some instructors consistently introduced the tracker during week 1, demonstrated its features, and checked usage during sessions. Others provided minimal explanation due to time constraints or uncertainty about the device. Participants reported inconsistent messaging, with some receiving detailed guidance and others receiving only basic setup instructions.

#### Barriers to Implementation

Key barriers to implementation included limited staff training on device features, competing demands during onboarding sessions, technical issues (eg, syncing and account access), and variability in participants’ digital literacy.

#### Facilitators of Implementation

Facilitators of implementation included staff enthusiasm, participants’ curiosity and motivation, clear visual feedback from the device, and integration with weekly physical activity sessions.

These findings indicate that implementation was feasible but uneven, with fidelity dependent on staff confidence and available time.

### Maintenance

The mean number of sessions all Weigh to Go participants attended during the maintenance phase of the Weigh to Go intervention was 1.91 (SD 2.57). Only 1 participant attended the full 15 weeks of the active and maintenance phases of the intervention.

Only 5 Weigh to Go participants provided Fitbit data beyond 20 weeks. The mean number of maintenance sessions attended by these 5 participants was 12.2 (SD 3.47).

#### Individual-Level Maintenance

Participants described mixed intentions to continue using the tracker after the program. Some planned to maintain daily step goals, others anticipated reduced use without structured support, and several noted that the device became less motivating once novelty faded.

The decline in MVPA at weeks 15 and 20 supports these qualitative accounts, indicating challenges in sustaining engagement without ongoing program structure.

#### Setting-Level Maintenance

Staff expressed interest in continuing to use trackers but highlighted the need for a formal protocol, clearer guidance on data use, consideration of long-term device costs, and integration into routine monitoring systems.

These findings suggest that maintenance at the organizational level would require structural support, resource allocation, and clearer processes.

### Qualitative Findings

Abductive thematic analysis of interviews conducted with the Weigh to Go participants (n=10) and health care professionals (n=10) involved in the delivery and onboarding of the Weigh to Go intervention identified 6 main themes and 11 subthemes. The themes are provided in Table 6 and discussed in more detail below.

**Table 6.** Study main themes (n=5) and subthemes (n=11).

Main themes	Subthemes
Fitbit use	1.1 Barriers 1.2 Enablers
Promotion	2.1 Health care 2.2 Weigh to Go intervention
Clinical care	3.1 Benefits of activity tracker use 3.2 Use by health care staff
Finance	4.1 Costs (patient and health care provider) 4.2 Health care monetary savings
Fitbit functions	5.1 Preferences 5.2 Interpretation
Effectiveness	Physical activity Goal setting

#### Fitbit Use

Participants described several barriers that limited their ability or willingness to use activity trackers. A central issue was low motivation to engage in physical activity, which diminished the perceived utility of the device. One participant reflected:

*I am not interested in exercising even though I know it is good for me. I just don’t have the drive to exercise.*

[Female participant, 62 years]

Such accounts illustrate that, while Fitbit devices can support behavior change, they are unlikely to generate motivation where this is fundamentally absent.

A further barrier related to digital literacy is particularly prevalent among older adults. As 1 health care professional noted:

*I think some of the older participants will struggle using activity trackers as they have poorer technology skills compared with the younger generation*.

[Female professional, 38 years]

This concern highlights the persistence of a digital divide that risks excluding certain groups unless targeted support is provided.

Financial constraints also emerged as a significant limitation. One professional commented:

*I know that some of those taking part in the Weigh to Go programme have little money and could not afford a Fitbit and do not own a mobile phone because of the cost.*

[Female professional, 47 years]

This illustrates that cost-related barriers extend beyond the device itself to include the ownership of compatible technologies and ongoing data costs.

For others, health conditions imposed practical limitations on activity tracker use. A participant with multiple chronic conditions explained:

*I have underlying health conditions such as arthritis and type 2 diabetes. These make it difficult to exercise and my mobility is poor.*

[Female participant, 76 years]

In such cases, the issue is not a lack of awareness or willingness, but the incompatibility of technological prompts with physical capacity.

Finally, participants reported that self-efficacy shaped their willingness to use a Fitbit. One male participant expressed:

*My confidence is very low due to my weight problems. This not only stops me exercising in public but it impacts other areas of my life like learning new skills.*

[Male participant, 58 years]

This suggests that psychological and social barriers, such as low confidence and fear of judgment, can limit both physical activity and engagement with new technologies.

Despite these barriers, participants identified several factors that could encourage uptake. Provision of a free device was repeatedly highlighted as a strong motivator:

*Normally I would not have bought a Fitbit but having had the device provided free has helped motivate me to be more active.*

[Female participant, 56 years]

Device provision at registration was seen as a way to reduce cost-related inequalities and promote engagement from the outset.

Participants also emphasized the importance of education and support. One explained:

*I wasn’t sure how best to use the Fitbit. I think a class dedicated on how best to use the Fitbit would have been very useful.*

[Female participant, 54 years]

Similarly, health care professionals highlighted the value of hands-on assistance:

*A number of your study participants needed support to set up the Fitbit device… I could help participants set up their trackers if required.*

[Male professional, 34 years]

These accounts underscore the importance of embedding structured technical support into interventions.

Goal setting was another key enabler. A professional suggested:

*As part of the Weigh to Go programme we could speak to each participant and set them physical activity goals over the 15 weeks.*

[Female professional, 41 years]

This reflects established evidence that personalized and incremental goal-setting strategies can enhance motivation and sustain behavior change.

#### Promotion

Health care professionals and participants proposed several mechanisms for promoting Fitbit use within clinical settings. The most direct approach was integration into consultations:

*This could be promoted through our practice or during a consultation*

[Female professional, 41 years]

Others suggested passive promotion through printed materials displayed in clinical environments:

*The NHS could produce posters, leaflets and handout documents within their premises or other public buildings.*

[Male professional, 34 years]

Finally, national-level campaigns were seen as potentially effective for raising awareness, with 1 participant proposing:

*A national campaign in local newspapers informing patients of this service might work.*

[Male participant, 41 years]

Within the Weigh to Go program, promotion at the point of registration was viewed as crucial:

*I was informed of this study when I first registered for the Weigh to Go programme. A friend of mine attended a different venue but was not told of this. I think promoting this at each venue would be a good idea.*

[Female participant, 69 years]

Beyond clinical settings, community spaces were highlighted as useful sites for engagement:

*I attend a companion group at my local community center each week… This would be a good place to promote the Fitbit.*

[Female participant, 66 years]

Such suggestions reflect the importance of both formal and informal networks in raising awareness.

#### Clinical Care

Participants emphasized the potential for Fitbit devices to contribute to clinical care. A health care professional highlighted:

*Using an activity tracker could form part of a patient’s care and if properly managed could improve their physical activity levels and improve their health.*

[Male professional, 34 years]

This was reinforced by participants who valued the potential for professional monitoring:

*The Fitbit could be used by doctor or nurse to monitor my activity and give me advice and exercise support if required.*

[Female participant, 30 years]

Health care professionals also considered the possibility of prescribing Fitbit devices, akin to medication or medical equipment:

*Activity trackers could be prescribed by say my doctor…*

[Female participant, 56 years]

Remote monitoring was seen as particularly advantageous:

*These pieces of technology would allow for the remote monitoring of a patient through their activity data and subsequently provide appropriate advice and support.*

[Female professional, 51 years]

Referral pathways were also considered, with 1 professional suggesting:

*If a patient is not undertaking enough exercise through analysis of their Fitbit information we could refer them to a local gym.*

[Male professional, 34 years]

#### Finance

Participants highlighted potential costs for both patients and providers. For patients, the primary expense was the purchase of the Fitbit itself:

*For me the main cost would be purchasing the Fitbit device.*

[Female participant, 48 years]

Others noted that the use of Fitbits necessitated additional costs such as smartphones and internet access:

*To use the Fitbit I would need to pay for internet access and to download the Fitbit app have a mobile phone.*

[Female participant, 60 years]

For health care providers, resource implications extended beyond devices to include staff training and time:

*The main costs for the health care provider beyond the price of the Fitbit would be staff training and staff time.*

[Female professional, 47 years]

Conversely, participants suggested that investment in activity trackers could generate long-term savings through improved health outcomes. One professional explained:

*If patients increase their physical activity and reduce their sedentary behavior the knock-on effect will be improved health and a reduction in the risk of developing future illnesses.*

[Male professional, 34 years]

Another emphasized reduced reliance on costly treatments:

*The healthier patients are then they will need less medications and expensive treatments.*

[Female professional, 51 years]

#### Fitbit Functions

Step counting was the most commonly used and valued feature, as 1 participant explained:

*I tended to only use the step function on the Fitbit as I found this easiest to understand.*

[Female participant, 56 years]

Other participants valued the ability to track weight:

*My Weigh to Go instructor told me how to type my weight into the Fitbit. This allowed me to monitor it while I was on the course.*

[Female participant, 30 years]

However, several participants described difficulties interpreting the full range of data provided by the device. One commented:

*I found the Fitbit motivational but other than steps I did not understand much of the information on the app.*

[Female participant, 76 years]

This was seen as a missed opportunity, with others suggesting that staff-led educational sessions could bridge this gap:

*I would have found it useful if our Weigh to Go class instructor had delivered a session explaining how best to use the Fitbit and how to interpret the information recorded.*

[Male participant, 41 years]

#### Effectiveness (Qualitative)

Participants generally perceived Fitbit devices as effective in increasing awareness of their physical activity levels and progress. For example:

*I enjoyed using the Fitbit. It helped me see any improvements in my activity levels*

[Female participant, 48 years]

The device also acted as a motivational prompt:

*I found the Fitbit motivated me to be more active and encourage me to do something on days when I felt tired.*

[Female participant, 60 years]

Health care professionals suggested that activity trackers could be integrated into structured goal-setting strategies within interventions such as Weigh to Go. As 1 professional argued:

*These devices could certainly be incorporated into the Weigh to Go programme and be used as an effective goal setting tool.*

[Male professional, 34 years]

### Summary

Overall, the findings highlight both the opportunities and challenges associated with incorporating Fitbit devices into weight management and clinical care. While barriers such as cost, digital literacy, and health conditions were evident, enablers such as device provision, education, and personalized goal-setting enhanced engagement. Promotion strategies at both clinical and community levels were proposed, and participants recognized the potential for activity trackers to support both behavior change and clinical monitoring. However, realizing these benefits will require attention to structural inequalities and adequate resourcing to ensure equitable access and effective integration.

## Discussion

### Principal Findings

This discussion reports a pragmatic evaluation of integrating a consumer activity tracker (Fitbit Charge 5) into Weigh to Go, a community weight management program delivered across Lanarkshire, Scotland. The purpose was not to test the effectiveness of Fitbit devices per se, but to examine feasibility, acceptability, and implementation issues in a real-world service using the RE-AIM framework [29,39]. Quantitative (Fitbit steps and MVPA minutes) and qualitative (semistructured interviews with Weigh to Go participants and health care professionals) data were integrated using an abductive approach to illuminate why the observed patterns occurred and what they imply for future scale-up [24-27,37].

The quantitative findings demonstrated an increase in daily steps and MVPA during the structured active phase of the program, followed by a decline during the maintenance phase. These patterns reflect the degree to which the tracker was embedded within the program structure: integration was strongest when supported by weekly sessions, staff engagement, and routine accountability, and weaker when participants were expected to self- manage.

The qualitative findings provided insight into how participants and staff experienced the tracker. Participants valued the immediate feedback, goal-setting features, and sense of accountability provided by the device. Staff recognized the potential of trackers to enhance engagement but reported variability in confidence, training, and time available to support device use.

#### Reach

Consistent with RE-AIM, reach concerns the absolute number, proportion, and representativeness of individuals who participate [29]. Seventy-one adults consented between July 2023 and January 2024; 59 were enrolled, and 33 contributed analyzable device data, with participants predominantly female and White and with a mean age in the mid-50s. Although 705 participants attended the targeted Weigh to Go venues during the recruitment window, the program did not record how many were approached, constraining precise calculation of recruitment rate and representativeness. Based on venue attendance, a conservative estimate suggests that approximately 10% of potentially eligible attendees consented (ie, 71/705), but this cannot be taken as a true denominator because approach rates were not logged. Future evaluations should prospectively track the recruitment path and report reasons for nonparticipation and attrition, in line with RE-AIM and CONSORT (Consolidated Standards of Reporting Trials) style flow logic adapted for pragmatic studies [39].

Qualitatively, participants and health care professionals emphasized that providing devices at no cost enhanced reach, particularly among people facing financial constraints and among men and ethnic minority groups, who are often underrepresented in weight management services [16,17]. However, digital access and digital literacy remained barriers for some older adults and those with limited technology confidence, echoing persistent “digital divide” concerns in community interventions [42].

#### Effectiveness

Within RE-AIM, effectiveness refers to impact on key outcomes, including potential harms. The within-subject analyses indicated a significant increase in daily steps between week 1 and week 7 (Bonferroni-adjusted, *P*=.008), with an average change of >5345 steps (reporting group means), whereas no statistically significant differences were observed for the other pairwise comparisons of steps or for weekly MVPA minutes over the same period. These results are presented cautiously as the study design does not permit causal attribution to the tracker or to any single component of Weigh to Go. Step count increases could reflect early engagement with the program as a whole, seasonal/contextual influences, or novelty effects [43], not necessarily the addition of a Fitbit device.

There was a difference between the recorded change in steps and MVPA. However, step counts often increase through additional light-intensity walking and incidental movement (eg, breaks in sedentary time), whereas MVPA requires higher-intensity bouts of physical activity [5,44]. In our qualitative data, participants described finding steps easy to understand and act upon, while feeling unsure how to interpret intensity metrics, suggesting an opportunity for education sessions focused on translating device feedback (active minutes and heart rate zones) into actionable weekly plans. Importantly, the Fitbit served simultaneously as an intervention component and measurement tool, a limitation we acknowledge below, given concerns about construct overlap and potential reactivity [10].

To improve interpretability in future phases, it is recommended that a single family of follow-up contrasts is used, either (1) stepwise (weeks 1-7, 7-15, and 15-20) or (2) baseline-referenced (week 1 vs weeks 7, 15, and 20) with appropriate multiplicity control (eg, Bonferroni or Holm). In this evaluation, the baseline-referenced approach was chosen to avoid mixing strategies, and weeks 1, 7, 15, and 20 were selected a priori to represent early engagement (week 1), midactive phase (week 7), end of active phase (week 15), and early maintenance (week 20), aligning with the program’s delivery structure.

Rather than evaluating program efficacy, our findings illustrate how the tracker functioned as an integrated component of the intervention. The observed increases in activity reflect how participants engaged with the device within the program context, while the later decline highlights the limits of integration when structured support is reduced.

#### Adoption

Adoption addresses the proportion and representativeness of settings and staff willing to initiate the intervention [29]. Across the service, 22 instructors and additional managers/administrators are engaged in Weigh to Go delivery, with 12 community venues used during the study period (community centers and fitness centers). Staff interviews revealed high conceptual openness to tracker integration but practical uncertainties regarding what exactly to do with device data, how to interpret dashboards meaningfully, and how to troubleshoot common issues (eg, syncing and account management). Although Fitbit devices are consumer-friendly, clinical/community adoption requires confidence in data interpretation, behavior change coaching with device metrics, and pathways for acting on data (eg, tailoring targets and referrals to supervised activity). This aligns with prior literature that wearables can support self-monitoring and feedback, but do not drive behavior change in isolation; professional guidance and integration into a behavioral program are essential [12,45,46].

At the organizational level, adoption is more likely where there is a protocol specifying eligibility, onboarding, data governance, coaching scripts, troubleshooting trees, and criteria for progression and referral (eg, to gym-based supervision). The interviewees explicitly requested such a protocol, which would also facilitate staff training and quality assurance across venues.

Staff adoption varied across roles and settings. Some instructors embraced the tracker as a motivational tool, while others viewed it as an optional add-on. Organizational adoption was influenced by staff training, perceived relevance, and available time. These findings highlight the importance of organizational readiness and clear role expectations when integrating digital tools into community programs.

#### Implementation

Implementation refers to fidelity, adaptations, costs, and practicalities [29]. The program delivered a 15-week active phase (education and exercise) followed by an optional 15-week maintenance phase. In practice, attendance during the active phase averaged 11.35 (SD 3.63) sessions, while maintenance attendance averaged 1.91 (SD 2.57) sessions. Only 1 participant attended all 30 sessions across active and maintenance phases; 5 submitted Fitbit data beyond 20 weeks. Interviews pointed to competing life demands, waning novelty, device/app literacy challenges, and variable goal-setting support as proximate reasons for drift. These reasons mirror broader evidence that self-monitoring adherence decays without ongoing reinforcement, social support, and personally meaningful goal setting [46-48].

From an economic standpoint, Weigh to Go’s reported average delivery cost was US $90.22 per participant for the 15-week active phase and US $180.44 for 30 weeks (active and maintenance), exclusive of devices. The retail cost of a Fitbit Charge 5 was US $136.30 during the study. A simple per-participant estimate for an activity tracker–enabled 15-week program is therefore approximately US $226.60 (program delivery plus device), rising with staff training and technical support. Venue costs were US $2707.65 per venue annually. Scaling to 125 venues at current venue costs implies approximately US $338,456.16 per year for venues alone, plus session delivery (eg, US $1805.10 per 30-week cycle per venue), devices, training, and support infrastructure. Given low maintenance attendance and data incompleteness, such scaling is unlikely to be cost-effective unless adherence can be substantially improved and unit costs reduced (eg, negotiated device pricing, shared equipment pools, or bring-your-own device models with equity safeguards). Prior work suggests that digital components can be cost-effective at scale only when engagement is sustained [49,50].

Two further implementation issues require explicit acknowledgment. First, measurement/intervention confounding arises when the Fitbit is used simultaneously as both the behavioral intervention component (ie, providing feedback, reminders, or activity prompts) and the outcome assessor (ie, recording steps, activity minutes, or heart rate). This dual role introduces a risk of reactivity, whereby participants may alter their behavior simply because they know their activity is being monitored by the device rather than due to the intended intervention mechanisms. In addition, relying on a single tool for both intervention delivery and measurement raises common method bias concerns, as the observed changes in outcomes may partly reflect the measurement method itself rather than genuine behavioral change [51]. Consequently, improvements recorded in physical activity metrics could be artificially inflated, undermining the ability to disentangle whether changes are attributable to the intervention’s efficacy, participants’ awareness of being monitored, or inherent limitations in the measurement device. Second, data loss occurred. Missing device data arose primarily from account and syncing errors (Google/Fitbit credential issues and intermittent app–phone connectivity), compounded by irregular wear. These problems were exacerbated by external vendor-driven changes, such as Google/Fitbit account migration, which caused unexpected authentication barriers. Syncing failures meant that even when devices were worn, data often did not upload consistently, while irregular wear patterns, charging lapses, and user disengagement further inflated missingness. Such losses created a disproportionate burden on staff, who had to provide troubleshooting, technical support, and device replacements, increasing hidden resource demands. Beyond logistical challenges, these gaps undermined analytic validity; data were systematically missing from lower-engagement participants, limiting statistical power and biasing outcome estimates. Together, these issues highlight that consumer devices, while seemingly low cost, introduce significant risks to data completeness, program fidelity, and equity unless robust technical and contractual safeguards are in place.

Implementation fidelity varied across sites. Some staff consistently introduced and supported tracker use, while others provided minimal guidance. Barriers included limited training, competing demands during onboarding, and variability in participants’ digital literacy. These findings underscore the need for structured implementation protocols, staff training, and dedicated time for device support.

#### Maintenance

Maintenance concerns sustained individual behavior change and the institutionalization of the program [29]. At the individual level, the sharp fall-off after the active phase indicates that behavioral maintenance was weak. Interviews suggest that goal progression, habit formation, and social accountability mechanisms were insufficiently embedded. Evidence indicates that maintenance benefits from graduated goals, implementation intentions, prompted self-regulation, and structured social support [8,19,48]. At the organizational level, maintenance would require a codified protocol, staff capability for data-guided coaching, and routine use of trackers in referral pathways (eg, condition-appropriate activity prescriptions, remote monitoring, or peer groups), as suggested by both participants and health care professionals and supported by remote monitoring literature [13].

Maintenance was limited at both the individual and organizational levels. Participants reported reduced motivation once the structured phase ended, and staff highlighted the need for clearer protocols, long-term funding, and integration with existing monitoring systems. These findings suggest that sustained integration requires ongoing support, resource allocation, and alignment with organizational processes.

### Consideration of Participant With Learning Difficulties

During the study, 1 Weigh to Go participant was identified as having learning difficulties through the demographics survey administered via Qualtrics. Ethical and methodological considerations were taken into account to ensure that their inclusion in the research was appropriate, respectful, and did not compromise the quality of the data collected.

For this participant, support was offered, and a member of the research team was available to provide clarification and to check comprehension at each stage.

Data collection procedures were also adjusted to accommodate the participants’ needs. Instructions for the use of the activity tracker were delivered slowly, repeated where necessary, and supplemented with practical demonstrations from venue staff. Written guidance was supported by verbal explanations. Regular check-ins were conducted to ensure that the device was being worn correctly and that data were being recorded as intended.

Ethically, it is important to reflect on the implications of including a participant with learning difficulties. Their participation demonstrates the inclusivity of the study design and highlights the feasibility of using wearable technologies across a broader range of populations, including those with additional support needs. It also underlines the importance of tailoring recruitment and data collection procedures to individual capabilities to maximize both participant experience and data quality.

Future research should more systematically address accessibility and inclusivity, ensuring that interventions involving wearable technologies are designed with flexibility to accommodate individuals with varying levels of literacy, cognitive capacity, and health literacy. By doing so, activity tracker interventions can be made more equitable and representative of real-world populations.

In summary, the study accounted for the needs of a participant with learning difficulties by providing adapted materials, additional support during consent and data collection, and regular check-ins to ensure compliance. Reflection on this inclusion highlights the importance of designing interventions and research methodologies that are accessible to diverse populations, which represents an important area for future development in this field.

### The Fitbit as Both Measurement Tool and Intervention Component

A key methodological consideration is that the Fitbit acted simultaneously as both a measurement tool and an intervention component. Its feedback, goal-setting, and reminder features inherently influence behavior, meaning that measurement and intervention effects cannot be fully disentangled. Recognizing this dual role is essential for interpreting outcomes and designing future studies that aim to isolate behavioral effects from device-driven reactivity.

### Strengths and Limitations

A key strength of this study is its pragmatic design, which examined the integration of a consumer activity tracker into an existing community-based weight management program (Weigh to Go) under real-world service conditions. By embedding the evaluation within a routine community intervention, findings reflect implementation realities, enhancing ecological validity and the relevance of recommendations for service delivery [39]. The use of the RE-AIM framework further strengthened the study by structuring the analysis across RE-AIM domains, facilitating a comprehensive evaluation of both individual and system-level outcomes [29].

Another strength lies in the mixed methods approach, which combined quantitative Fitbit-derived data with qualitative interviews from both Weigh to Go participants and health care professionals. This methodological triangulation provided richer insight into not only whether physical activity increased but also how and why adoption and engagement occurred [24]. The abductive analytic strategy enabled a nuanced interpretation that linked empirical findings with theoretical concepts, capturing unanticipated patterns such as the divergence between step count improvements and minimal changes in moderate-to-vigorous–intensity physical activity [26,31].

The study also benefited from the inclusion of multiple stakeholders, with perspectives gathered from Weigh to Go participants and health care professionals involved in program delivery. This broadened the analysis beyond individual behavior change to include service delivery challenges, data governance considerations, and resource implications, which are critical elements for informing scale-up [46]. Furthermore, the provision of free Fitbit devices increased participation among individuals who might otherwise face financial barriers, supporting equity of access and contributing to insights around reach [16].

A major strength of this study is its real- world context, which enhances ecological validity. The mixed methods design allowed for a nuanced understanding of integration across RE-AIM dimensions. However, substantial Fitbit data loss limited the representativeness of quantitative findings. This limitation is best understood not only as a data quality issue but also as an indicator of integration challenges, particularly around account management, technical support, and participant responsiveness.

Despite these strengths, several limitations must be acknowledged. First, the noncontrolled design precludes causal inference. Increases in daily step counts, particularly between week 1 and week 7, cannot be attributed solely to Fitbit use, as changes may also reflect general engagement with the Weigh to Go program, seasonal influences, or novelty effects [43]. Second, the Fitbit device functioned both as an intervention and as the primary measurement tool, raising concerns about measurement reactivity, construct overlap, and reliance on proprietary algorithms [10,51].

Third, data completeness and quality issues emerged, with missing Fitbit records due to syncing errors, account access problems, and irregular device wear. Such missingness may bias estimates and reduce the statistical power to detect changes. Fourth, maintenance engagement was low, with very few participants providing data beyond 20 weeks and only 1 completing the full 30-week program. This point was not explored during the qualitative interviews, particularly with the participant who completed the full 30-week program. Overall, these limitations restrict conclusions regarding long-term sustainability, which is critical for population-level type 2 diabetes prevention [48].

Fifth, the sample lacked sociodemographic diversity. The participant group was predominantly White, female, and middle-aged, reducing the representativeness of findings and limiting generalizability to more diverse populations, including men, younger adults, and ethnic minority groups. Given evidence of disparities in both type 2 diabetes prevalence and digital health engagement, future studies should prioritize targeted recruitment and culturally tailored strategies [51].

Finally, while the study provided illustrative economic estimates, these relied on assumptions regarding device costs, staff time, and venue use. A full economic evaluation incorporating cost-effectiveness modeling and sensitivity analyses is needed to assess scalability and sustainability [49,50].

In summary, this study provides valuable real-world evidence on the integration of consumer activity trackers into a community weight management program, highlighting both opportunities and challenges. Its strengths lie in its pragmatic, mixed methods design and stakeholder inclusion, while its limitations include design constraints, data loss, limited diversity, and uncertain long-term outcomes. These considerations should inform future controlled evaluations, independent measurement strategies, and implementation planning.

### Clarification of Pragmatic Evaluation

We describe this study as a pragmatic evaluation to emphasize that it examined how the tracker functioned within a real-world service context, rather than testing the efficacy of the Weigh to Go program under controlled conditions. This does not imply a change in study design but clarifies the interpretive lens applied to the findings.

### Implications and Conclusions

Within a real-world community program, integrating an activity tracker is feasible and perceived as acceptable by many participants and staff. However, sustained engagement is the principal challenge and a determinant of value for money. The quantitative signal of increased steps early in the active phase, without corresponding MVPA change, is consistent with light-intensity activity displacement rather than structured intensity gains. Education on interpreting intensity metrics, goal progression, and coaching appears necessary to shift MVPA. To support adoption and implementation at scale, the service should develop a formal protocol covering onboarding, data governance, coaching scripts, and referral triggers, deliver role-specific staff training on data interpretation, troubleshooting, and behavior-change techniques, and strengthen maintenance via scheduled check-ins, social/peer support, graduated goals, and remote prompts/feedback. Economically, large-scale roll-out (eg, 125 venues) will remain fragile unless adherence improves and unit costs, notably device and support, are reduced through procurement and design efficiencies.

Reframed as a pragmatic evaluation rather than an efficacy test, this study contributes evidence on how and under what conditions activity trackers can be integrated into community weight-management services for adults at risk of or living with type 2 diabetes.

Future research should include comparative designs (eg, stepped-wedge or cluster randomized control trials), independent activity assessment (accelerometry), and economic evaluation, coupled with qualitative process evaluations grounded in reflexive thematic analysis to explain mechanisms and context. Only with stronger maintenance supports and clear operational protocols is tracker-enabled scale-up likely to achieve meaningful population health. A key methodological insight is that the Fitbit acted simultaneously as a measurement tool and an intervention component. Its feedback and goal-setting features inherently influence behavior, meaning that measurement and intervention effects cannot be fully separated. Future research should consider designs that explicitly address this dual role.

## Abbreviations

CONSORT: Consolidated Standards of Reporting Trials
COREQ: Consolidated Criteria for Reporting Qualitative Research
MVPA: moderate-to-vigorous–intensity physical activity
RE-AIM: Reach, Effectiveness, Adoption, Implementation, and Maintenance
